# The measurement of consciousness: a framework for the scientific study of consciousness

**DOI:** 10.3389/fpsyg.2014.00714

**Published:** 2014-07-10

**Authors:** David Gamez

**Affiliations:** Sackler Centre for Consciousness Science / Department of Informatics, University of SussexBrighton, UK

**Keywords:** measurement, correlates, consciousness, causal closure, first-person report

## Abstract

Scientists studying consciousness are attempting to identify correlations between measurements of consciousness and the physical world. Consciousness can only be measured through first-person reports, which raises problems about the accuracy of first-person reports, the possibility of non-reportable consciousness and the causal closure of the physical world. Many of these issues could be resolved by assuming that consciousness is entirely physical or functional. However, this would sacrifice the theory-neutrality that is a key attraction of a correlates-based approach to the study of consciousness. This paper puts forward a different solution that uses a framework of definitions and assumptions to explain how consciousness can be measured. This addresses the problems associated with first-person reports and avoids the issues with the causal closure of the physical world. This framework is compatible with most of the current theories of consciousness and it leads to a distinction between two types of correlates of consciousness.

## Introduction

Consciousness just is not the sort of thing that can be measured directly. What, then, do we do without a consciousness meter? How can the search go forward? How does all this experimental research proceed?

I think the answer is this: we get there with principles of *interpretation*, by which we interpret physical systems to judge the presence of consciousness. We might call these *preexperimental bridging principles*. They are the criteria that we bring to bear in looking at systems to say (1) whether or not they are conscious now, and (2) which information they are conscious of, and which they are not.

Chalmers (1998), p. 220

A science that invokes mental phenomena in its explanations is presumptively committed to their causal efficacy; for any phenomenon to have an explanatory role, its presence or absence in a given situation must make a difference – a *causal difference*.

Kim (1998), p. 31

Consciousness is a significant research topic in philosophy and elaborate thought experiments have been developed about the relationship between consciousness and the physical world. However, there is little agreement about the nature of consciousness and it can be argued that our theories have failed to advance much beyond Descartes. To address this impasse it has been proposed that we should use scientific experiments to identify correlations between consciousness and the physical world while suspending judgment about which metaphysical theory of consciousness (if any) is correct (Hohwy, 2007). When we have more detailed information about the relationship between consciousness and the physical world it might be possible to develop mathematical descriptions of this relationship that could be experimentally tested. More data about the correlates of consciousness could also help us to address philosophical questions about consciousness.

In an experiment on the correlates of consciousness we measure the state of the physical world, measure consciousness^1^, and look for spatiotemporal structures in the physical world that only occur whenever a particular conscious state is present. Consciousness can only be measured through first person reports, which raises questions about their accuracy, the potentially large variability in people's consciousness, and the possibility that there could be non-reportable consciousness. First-person reports also have physical effects, such as movement of body parts or vibrations in the air. Since consciousness is the putative cause of these reports, it presumably has to be the sort of “thing” that can bring about changes in the physical world. While this does not present a problem for reductionist theories of consciousness, such as functionalism (Kim, 1998), reports from a non-physical consciousness would undermine the causal closure of the physical world. We are apparently forced to make functionalism or physicalism our working assumption if we want to measure consciousness. This would be disputed by many people and it sacrifices the theory neutrality that is a key attraction of a correlates-based approach to consciousness. A number of solutions have been put forward to this problem, including dynamical systems approaches (Van de Laar, 2006), causal overdetermination (Bennett, 2003; Kroedel, 2008), and intralevel causation (Buckareff, 2011). However, the issue remains extremely controversial, and each proposed solution is subject to its own difficulties and limitations.

This article suggests how this problem could be addressed by framing the scientific study of consciousness within a set of assumptions that explain how we can measure consciousness without getting entangled in debates about first-person reporting and the causal relationship between consciousness and the physical world. This approach is compatible with most of the current theories of consciousness and it is similar in intent to Chalmers' (1998) pre-experimental bridging principles, although it differs considerably in the details.

The framework of assumptions that is presented in this paper is designed to insulate the scientific study of consciousness from philosophical problems, such as zombies, color inversion and the causal relationship between consciousness and the physical world. Most scientific research on consciousness does not directly engage with these problems, but they are valid concerns that could potentially jeopardize experimental results. It is unlikely that these problems can be easily solved, but it is possible to make a reasonable set of assumptions that explicitly set them aside. The results from the science of consciousness can then be considered to be true *given these assumptions*. For example, scientists cannot prove that their subjects are reporting all of their consciousness, but they can assume that unreportable consciousness is not present during experiments on the correlates of consciousness (assumption A4—see section Platinum Standard Systems). While this framework has important benefits for the scientific study of consciousness, it also constrains the theories of consciousness that can be put forward. For example, these assumptions are incompatible with Zeki and Bartels' (1999) proposal that micro-consciousnesses are distributed through the brain and they suggest that all correlates of consciousness have to be connected to first-person reports.

The first part of this paper gives an overview of the scientific study of consciousness and sets out a number of working assumptions about the relationship between consciousness and the normally functioning adult human brain. The next part puts forward an interpretation of the measurement of consciousness that does not rely on a premature commitment to functionalism or physicalism and which does not break the causal closure of the physical world. This has implications for experimental work on the correlates of consciousness and it leads to a division of proposed correlates of consciousness into two distinct types. Some implications of this approach for the science of consciousness are discussed in the last part and the complete set of definitions, lemmas, and assumptions is provided as an appendix to this paper.

## The science of consciousness

This section gives an overview of experiments on the correlates of consciousness, which measure consciousness, measure the physical world and look for spatiotemporal structures that are correlated with conscious states. A number of assumptions are needed to handle the fact that a brain's consciousness can only be measured indirectly through first person reports, which can also be generated by systems that are not typically thought to be conscious, such as computers. It is also necessary to assume that consciousness cannot vary independently of our measurement of it, which would undermine our ability to study consciousness scientifically.

### Measurement of consciousness (c-reports)

A full discussion of the best way to define consciousness is beyond the scope of this article. The working definition that I will use is that consciousness is the stream of experience that appears when we wake up in the morning and disappears when we fall into deep sleep at night. This can have different levels of intensity (from drowsy to hyper alert) and a wide variety of contents. We cannot directly detect the consciousness of another person, and so a variety of external behaviors are used to infer the presence of conscious states.

When I say “I am conscious” I am stating that I can see objects distributed in space around me, that I can hear, smell and touch these objects and attend to different aspects of them. A report of a conscious experience can be spoken, written down, or expressed as a set of responses to yes/no questions—for example, when patients communicate by imagining playing tennis or walking around a house in an fMRI scanner (Monti et al., 2010)^2^. People can be asked to subjectively assess the clarity of their visual experience (Ramsøy and Overgaard, 2004), and their level of awareness of a stimulus can be extracted using indirect measures, such as post-decision wagering (Persaud et al., 2007)^3^.

When people are not explicitly reporting their consciousness they can still be considered to be conscious on the basis of their external behavior. For example, Shanahan (2010) has argued that enhanced flexibility in the face of novelty and the ability to inwardly execute a sequence of problem-solving steps are a sign of consciousness, and the Glasgow Coma Scale uses motor responsiveness, verbal performance and eye opening to measure the level of consciousness in patients (Teasdale and Jennett, 1974). An overview of some of the different techniques for measuring consciousness is given by Seth et al. (2008).

I will use “c-report” to designate any form of external behavior that is interpreted as a report about the level and/or contents of consciousness. This paper will primarily focus on verbal c-reporting, on the assumption that similar arguments can be applied to any form of behavioral report about consciousness. C-reporting will be interpreted in the fullest possible sense, so that every possible detail of a conscious experience that could be reported will be assumed to be reported.

One of the key problems with c-reporting is that it is hard to obtain accurate detailed descriptions of conscious states. Consciousness changes several times per second and it is altered by the act of c-reporting, so how can we describe it using natural language, which operates on a time scale of seconds? Shanahan (2010) has suggested that this problem could be addressed by resetting our consciousness, so that multiple probes can be run on a single fixed state (see section Platinum Standard Systems). People can also be trained to make more accurate reports about their consciousness (Lutz et al., 2002), and there has been a substantial amount of work on the use of interviews to help people describe their conscious states^4^. These problems have led to a debate about the extent to which we can generate accurate descriptions of our consciousness (Hurlburt and Schwitzgebel, 2007).

C-reports are typically transformed into natural language descriptions of a state of consciousness. However, natural language is not ideal for describing consciousness because it is context-dependent, ambiguous and it cannot be used to describe the experiences of non-human systems (Chrisley, 1995). It is also difficult to see how natural language descriptions could be incorporated into mathematical theories of consciousness. One way of addressing these problems would be to use a tightly structured formal language to describe consciousness (Gamez, 2006). Chrisley (1995) has made some suggestions about how consciousness can be described using robotic systems, although it is not clear to what extent these proposals could be play a role in a mathematical theory of consciousness.

### Measurement of unconscious information (uc-reports)

The absence of a c-report about the level and/or contents of consciousness is typically taken as a sign that a person is unconscious or that a particular piece of information in the brain is unconscious. People can also make deliberate reports of unconscious mental content. For example, forced choice guessing is used in psychology experiments to measure unconscious mental content and visually guided reaching behavior in blindsight patients is interpreted as a sign that they have access to unconscious visual information. Galvanic skin responses can indicate that information is being processed unconsciously (Kotze and Moller, 1990) and priming effects can be used to determine if words are being processed unconsciously—for example, Merikle and Daneman (1996) played words to patients under general anesthesia and found that when they were awake they often completed word stems with words that they had heard unconsciously.

All of these types of unconscious reporting will be referred to as “uc-reports,” which are any form of positive or negative behavioral output that is interpreted as the absence of consciousness or the presence of unconscious information. While there will inevitably be gray areas between c-reports and uc-reports, it will be assumed that there are enough clear examples of both types to justify the distinction in this paper.

### Platinum standard systems

To scientifically study consciousness we need to start with a physical system that is commonly agreed to be capable of consciousness and whose c-reports can be believed to be about consciousness. The typical approach that is taken in empirical work on consciousness is to set aside philosophical worries about solipsism and zombies, and make the assumption that the human brain is capable of consciousness. This assumption can be made more general by introducing the notion of a platinum standard system, which is defined as follows ^5^:

**D1**. A platinum standard system is a physical system that is assumed to be associated with consciousness some or all of the time.

By “associated” it is meant that consciousness is linked to a platinum standard system, but no claims are being made about causation or metaphysical identity. With this definition in place, we can make the explicit assumption that the human brain is a platinum standard system^6^:

**A1**. The normally functioning adult human brain is a platinum standard system.

By “normally functioning” it is meant that the brain is alive, that it would be certified as normally functioning by a doctor, and that it does not contain any unusual chemicals that might affect its operation^7^. While the normally functioning adult human brain is currently the only system that is confidently associated with consciousness, further assumptions could be added to extend the number of platinum standard systems—for example, claiming that infant, monkey or alien brains are associated with consciousness.

A second issue in consciousness research is the possibility that two platinum standard systems in similar states could be associated with radically different consciousnesses while manifesting the same behavior. For example, there is the classic problem of color inversion, according to which I might experience red when my brain is in a particular state, you might experience green, and we could both use “blue” to describe our conscious states. More complicated situations can be imagined—for instance, my consciousness of having a bath could be remapped onto a behavioral output that controls an airplane. If consciousness can vary independently of the physical world, then it will be impossible to systematically study the relationship between consciousness and the physical world.

A simple way of addressing this issue is to assume that consciousness supervenes on the physical world. Since we are only concerned with developing a pragmatic approach to the science of consciousness, it is not necessary to assume that consciousness logically or metaphysically supervenes on the brain—we just need to assume that the natural laws are such that consciousness cannot vary independently of the physical world:

**A2**. The consciousness associated with a platinum standard system nomologically supervenes on the platinum standard system. In our current universe physically identical platinum standard systems are associated with identical consciousness.

The c-reports that are used to measure consciousness can be cross-checked against each other for consistency, but there is no ultimate way of establishing whether a set of c-reports from a platinum standard system correspond to the consciousness that is associated with the platinum standard system. Since c-reports are the only way in which consciousness can be scientifically measured, it has to be explicitly assumed that c-reports from a platinum standard system co-vary with its consciousness:

**A3**. During an experiment on the correlates of consciousness, the consciousness associated with a platinum standard system is functionally connected to its c-reports about consciousness.

A3 captures the idea that when we make a c-report about consciousness, what we say about consciousness has some correspondence with the consciousness that is being c-reported. The functional connectivity means that the link between consciousness and c-reports is a deviation from statistical independence, not a causal connection^8^. A3 does not specify the amount of functional connectivity between consciousness and the c-reports, which might be quite low because of the limits of the c-reporting methods. A3 is also explicitly restricted to experimental work, which leaves open the possibility that predictions could be made about consciousness in situations in which c-reporting is disconnected from consciousness.

A contrastive experiment that compares the states of the conscious and unconscious brain is meaningless if the apparently unconscious brain is actually conscious but unable to report or remember its consciousness. Similarly, a binocular rivalry experiment on consciousness is worthless if the apparently unconscious information is associated with a separate consciousness that is disconnected from the memory and/or reporting systems. Ghostly ecosystems of unreportable consciousnesses would completely undermine all contrastive experiments on consciousness—scientific studies can only proceed on the assumption that they do not exist:

**A4**. During an experiment on the correlates of consciousness all conscious states associated with a platinum standard system are available for c-reports about consciousness.

A4 assumes that all conscious states in a platinum standard system are available for c-report, even if they are not actually reported during an experiment^9^. This makes it possible to use a variety of c-reports to extract a complete picture of the consciousness associated with a particular state of a platinum standard system. To circumvent the problems of limited working memory it might be necessary to put the system into a particular state, run the probe, reset the system and apply a different probe, until all of the data about consciousness has been extracted^10^.

Assumption A4 is explicitly limited to experiments on the correlates of consciousness. During these experiments it is assumed that the consciousness that is present in the system can be measured, which is a condition of possibility for this type of experimental work. While phenomenal consciousness and access “consciousness” *might* be conceptually dissociable (Block, 1995)^11^, the idea that non-measureable phenomenal consciousness could be present during experiments on the correlates of consciousness is, from the perspective of this paper, incompatible with the scientific study of the correlates of consciousness. A4 is also incompatible with panpsychism, which claims that apparently unconscious parts of the brain and body are associated with an inaccessible consciousness. For similar reasons A4 is likely to be incompatible with Zeki and Bartels' (1999) proposal that micro-consciousnesses are distributed throughout the brain. Outside of experiments on the correlates of consciousness it is possible, even likely, that there could be inaccessible phenomenal consciousness. Information gathered by experiments on the correlates of consciousness could be used to make predictions about the presence of phenomenal consciousness in these situations—for example, it could be used to make predictions about consciousness in brain damaged patients, infants or animals.

### Correlations between consciousness and the physical world

In this paper, the correlates of consciousness are defined in a similar way to Chalmers' (2000) definition of the total correlates of consciousness^12^:

**D2**. A correlate of a conscious experience, *e*_1_, is a minimal set of one or more spatiotemporal structures in the physical world. This set is present when *e*_1_ is present and absent when *e*_1_ is absent.

**Figure 1:**
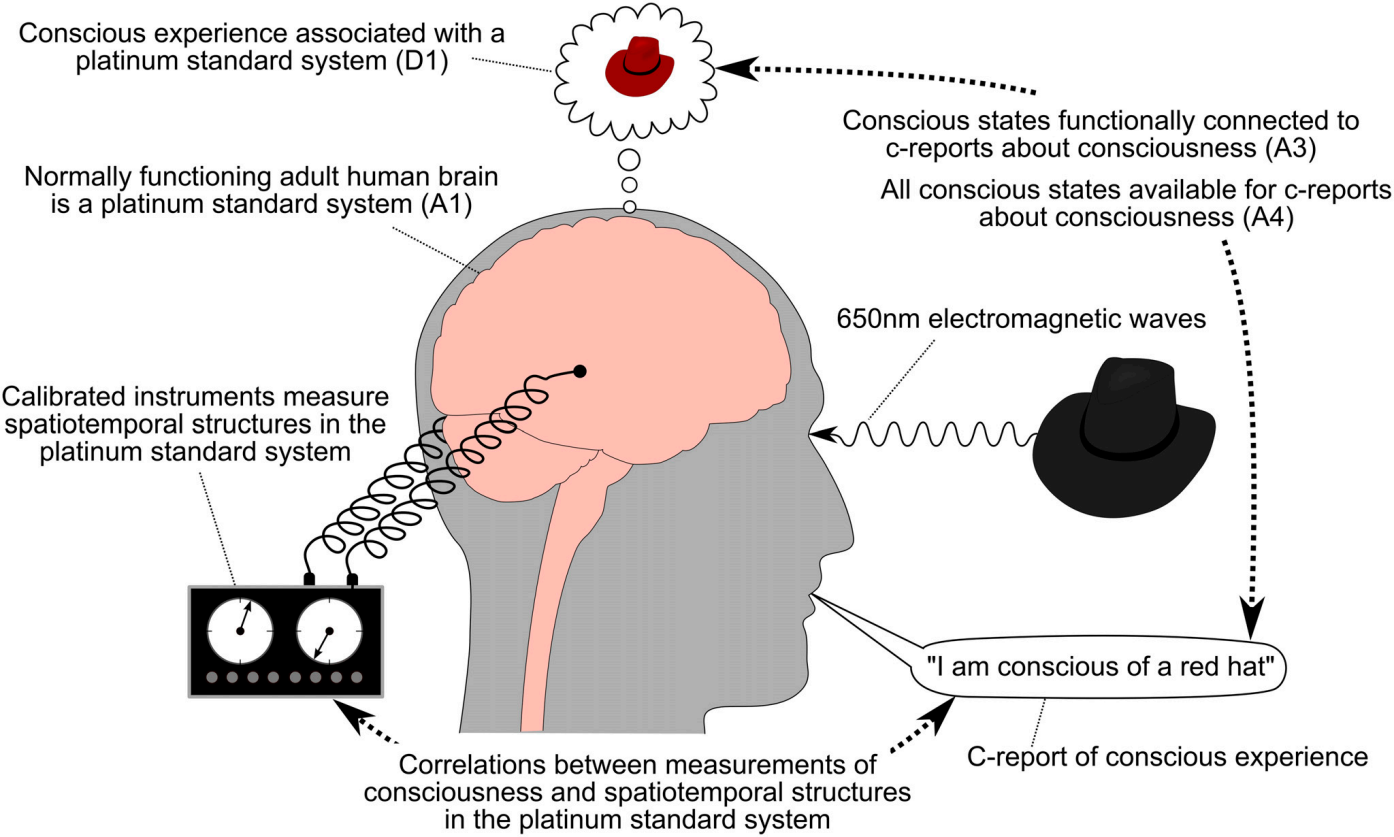
**Experiment on the correlates of consciousness**. The normally functioning adult human brain is assumed to be a platinum standard system that is associated with consciousness (D1 and A1). All of this system's conscious states are available for c-reports (A4), which are functionally connected to its conscious states (A3). Correlations are identified between the spatiotemporal structures in the platinum standard system and the c-reports about consciousness^14^.

The notion of a minimal set is intended to exclude features of a platinum standard system that typically occur at the same time as consciousness, but whose removal would not lead to the alteration or loss of consciousness. For example, the correlates of consciousness in the brain might have prerequisites and consequences (see section Separating out the Correlates of Consciousness) that would typically co-occur with consciousness, but the brain would be conscious in exactly the same way if the minimal set of correlates could be induced without these prerequisites and consequences. Correlates defined according to D2 would continue to be associated with consciousness if they were extracted from the brain or implemented in an artificial system. I have excluded terms like “necessity” and “sufficiency” from D2 because they could imply that the physical brain *causes* consciousness, which is not required for a strictly correlations-based approach^13^. “Spatiotemporal structures” is a deliberately vague term that captures anything that might be correlated with consciousness, such as activity in brain areas, neural synchronization, electromagnetic waves, quantum events, etc. The minimal set of spatiotemporal structures can be established by systematic experiments in which all possible combinations of candidate features are considered (see Table 1). An experiment on the correlates of consciousness is illustrated in Figure 1.

**Table 1:** **Illustrative example of correlations that could exist between conscious experiences (*e*_1_ and *e*_2_) and a physical system**.

**Spatiotemporal structures**				**Conscious experiences**	
**A**	**B**	**C**	**D**	***e_1_***	***e_2_***
0	0	0	0	0	0
0	0	0	1	0	0
0	0	1	0	0	1
0	0	1	1	0	1
0	1	0	0	0	0
0	1	0	1	0	0
0	1	1	0	0	1
0	1	1	1	0	1
1	0	0	0	0	0
1	0	0	1	0	0
1	0	1	0	0	1
1	0	1	1	0	1
1	1	0	0	1	0
1	1	0	1	1	0
1	1	1	0	1	1
1	1	1	1	1	1

*It is assumed that e_1_ and e_2_ can occur at the same time. A–D are spatiotemporal structures in a platinum standard system, such as dopamine, neural synchronization or 40 Hz electromagnetic waves. A–D are assumed to be the only possible features of the system. “1” indicates that a feature is present; “0” indicates that it is absent. In this example D is not a correlate of consciousness because it does not systematically co-vary with either of the conscious states. {A,B} is a set of spatiotemporal structures that correlates with conscious experience e_1_. {C} is a set of spatiotemporal structures that correlates with conscious experience e_2_*.

While there has been an extensive amount of work on the *neural* correlates of consciousness, it has not been demonstrated that consciousness is only correlated with activity in biological neurons. It is possible that spatiotemporal structures in other components of the brain, such as hemoglobin or glia, are correlated with consciousness as well. To fully understand the relationship between consciousness and the physical world we need to consider all possible spatiotemporal structures in a platinum standard system that might be correlated with consciousness (Gamez, 2012).

Definition D2 enables me to state assumption A2 more precisely:

**A2a**. The consciousness associated with a platinum standard system nomologically supervenes on the correlates of consciousness in the platinum standard system. In our current universe the spatiotemporal structures that correlate with conscious experience *e*_1_ will be associated with *e*_1_ wherever they are found.

Finally, since the correlates of consciousness are not statistically independent from a platinum standard system's consciousness, they can also be described as features of a platinum standard system that are *functionally connected* to its conscious states. This way of describing the relationship between consciousness and the physical brain will play a role in what follows, and so it will be formally stated as lemma 1:

**L1**. There is a functional connection between consciousness and the correlates of consciousness.

## Causation and reports about consciousness

The definitions, assumptions and lemmas that have been presented so far put us in a good position for the scientific study of consciousness. We have a set of systems that are capable of consciousness and their consciousness cannot vary independently of the physical world. C-reports can be used to measure consciousness, and all of a system's consciousness is available for c-report during an experiment on the correlates of consciousness.

This part of the paper addresses the question of how the measurement of consciousness relates to the causal closure of the physical world. The section on empirical causation develops a clearer understanding of physical causation, which is used to relate the framework that has been developed so far to causal relationships in the brain and world. This leads to a distinction between two types of correlates of consciousness and it clarifies how the correlates of consciousness can be experimentally separated out from other spatiotemporal structures in the brain.

### Empirical causation (e-causation)

Causal concepts play an important role in the philosophy of mind and claims are often made about the causal closure of the physical world and the causal impotency of mental states. These issues could be addressed more effectively if we had a clearer understanding of the nature of causation, but this a contentious topic and there is no generally agreed theory.

A first step towards a better understanding of causation is Dowe's (2000) distinction between a *conceptual* analysis of causation that elucidates how we understand and use causal concepts in our everyday speech, and an *empirical* account of causation, which explains how causation operates in the physical world^15^. Predominantly conceptual accounts of causation include Lewis' (1973) counterfactual analysis and Mackie's (1993) INUS conditions. Empirical theories reduce causation to the exchange of physically conserved quantities, such as energy and momentum (Aronson, 1971a,b; Fair, 1979; Dowe, 2000), or link causation with physical forces (Bigelow et al., 1988; Bigelow and Pargetter, 1990).

While conceptual analyses of causation remain popular within philosophy, it is difficult to see how our use of “causation” in everyday speech could help us to understand the causal interactions in the brain's neural networks and the relationship between consciousness and the physical world. Furthermore, some of the problems with the measurement of consciousness are linked to the causal closure of the physical world. This can be precisely defined using an empirical account of causation, but it is less clear how this can be done with a conceptual account. Other advantages of empirical theories include their ability to precisely identify causal events, to exclude cases of apparent causation between correlated events, and to easily relate the causal laws governing macro scale objects, such as cars and trees, to the micro scale interactions between molecules, atoms and quarks.

A detailed discussion of the advantages and disadvantages of different theories of empirical causation is beyond the scope of this paper, but it will be easier to analyze the c-reporting of consciousness with a concrete theory in mind. For this purpose I will use Dowe's theory of empirical causation, which is the most fully developed conserved quantities approach and has the following key features:

- A *conserved quantity* is a quantity governed by a conservation law, such as mass-energy, momentum or charge.
- A *causal process* is a world line of an object that possesses a conserved quantity.
- A *causal interaction* is an intersection of world lines that involves the exchange of a conserved quantity.

This account of causation will be referred to as *e-causation*. The framework developed in this paper relies on there being *some* workable theory of empirical causation, but it does not depend on the details of any particular account—if Dowe's theory is found to be problematic, an improved version can be substituted in its place. If all empirical approaches to causation turn out to be unworkable, then we might have to limit causal concepts to ordinary language and abandon the attempt to develop a scientific understanding of the causal relationship between consciousness and the physical world.

**Figure 2:**
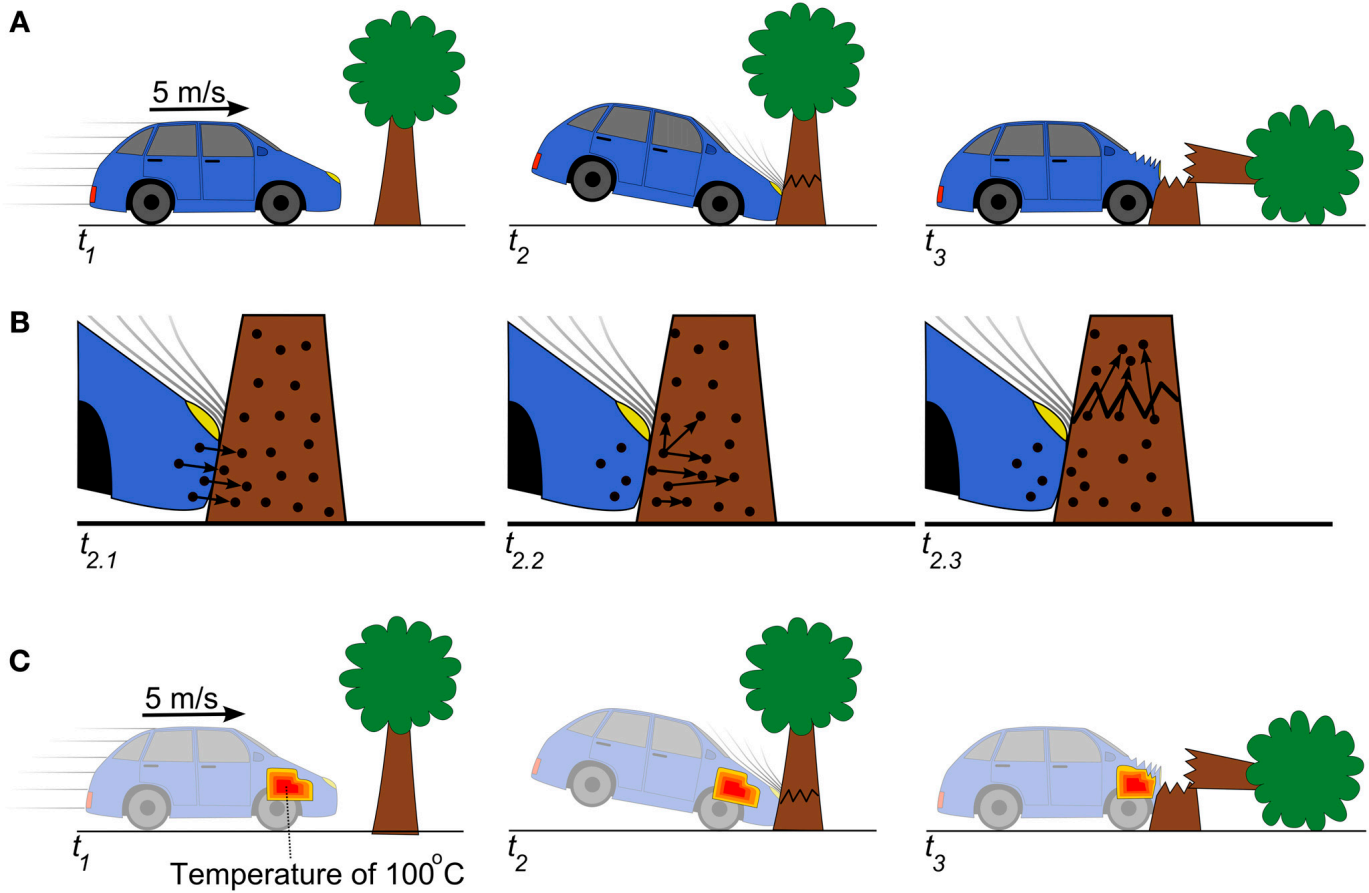
**The relationship between macro- and micro-scale e-causal events. (A)** A car moving at 5 m/s collides with a tree and the tree falls over. This is a macro-scale e-causal event in which the car passes energy-momentum to the tree. **(B)** The macro-scale e-causal interaction between the car and tree can be reduced to the micro-scale exchanges of energy-momentum between atoms in the car and tree. **(C)** The temperature of the car's engine is a macro-scale property that moves at 5 m/s and collides with the tree. The engine temperature exchanges a small amount of energy-momentum with the tree in the form of heat, but not enough to e-cause the tree to fall down.

To better understand how e-causation can explain causal relationships at different levels of description of a system, consider the example of a car moving along a road at 5 m/s that collides with a tree and knocks it over (Figure 2A). This is a clear example of an e-causal interaction between large scale objects in which physically conserved quantities are exchanged between the car and tree. This macro-scale e-causal interaction can be reduced down to the micro-scale e-causal interactions between the physical constituents of the car and tree (Figure 2B), and an empirical approach to causation also enables us to distinguish between true and false causes of a particular event. For example, the car's engine temperature is a macro-scale property of the physical world that moves along at the same speed as the car and collides with the tree (Figure 2C). However, the macro property of engine temperature does not exchange physically conserved quantities with the tree (ignoring any minor transfer of heat), and so the engine temperature does not e-cause the tree to fall down, although it can e-cause other macro-scale events, such as the melting of ice. Similar e-causal accounts can be given of the laws of other macro-scale sciences, such as geology, chemistry, and biology^16^.

In physics it is generally assumed that the amount of energy-momentum in the physical universe is constant as long as the reference frame of the observer remains unchanged—when part of the physical world gains energy-momentum, this energy-momentum must have come from elsewhere in the physical universe. It is also generally assumed that the net quantity of electric charge in the universe is conserved, so if part of the physical world gains electric charge, another part of the physical world must have lost charge or there must have been an interaction in which equal quantities of positive and negative charge were created or destroyed. Similar arguments apply to other physically conserved quantities, which leads to the following assumption:

**A5**. The physical world is e-causally closed.

According to A5, any change in a physical system's conserved quantities can in principle be traced back to a set of physical e-causes that led the system to gain or lose those conserved quantities at that time^17^.

### E-causation and reports about consciousness

**Figure 3:**
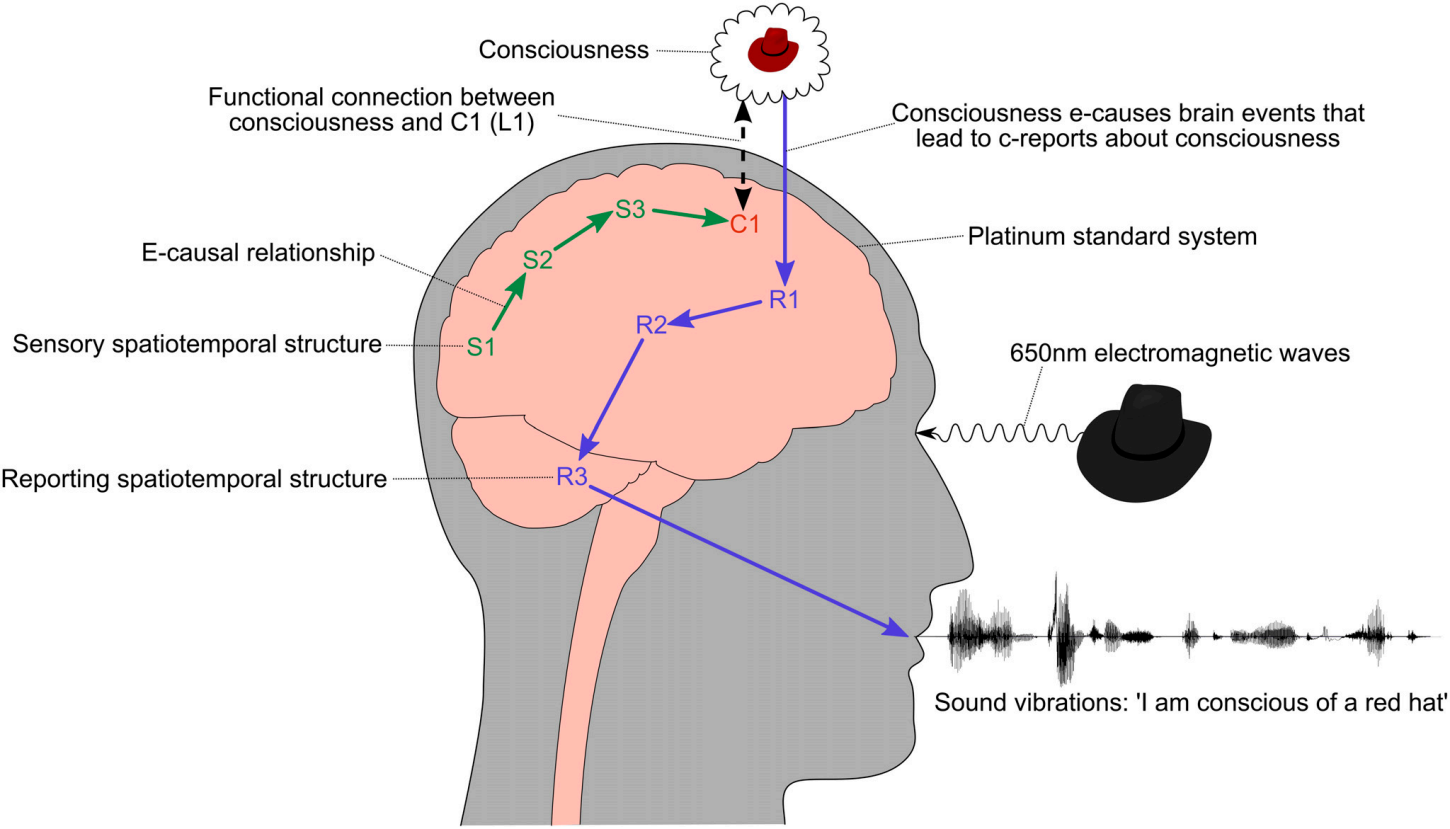
**Naive picture of how consciousness leads to a c-report about consciousness**. The labels S1, C1, R1, etc. refer to any kind of spatiotemporal structure in the brain, such as the activation of a brain area, neural synchronization, electromagnetic waves, quantum events, and so on. Their names and locations are only illustrative and not intended to correspond to particular anatomical paths or structures. An e-causal chain of sensory spatiotemporal structures, S1–S3, leads to the appearance of a spatiotemporal structure, C1, that is correlated with consciousness. In this example, the content of consciousness is determined by the sensory events, but in principle it could be independent of S1–S3, for example if the subject was dreaming. The conscious state is the beginning of an e-causal chain of spatiotemporal structures, R1–R3, that result in a verbal description of the consciousness.

C-reports about consciousness are changes in the physical world (vibrations in the air, marks on paper, button presses, movements of limbs, etc.) that enable people to gain information about each other's conscious states. In everyday language we speak about a person reporting their consciousness, describing their consciousness, and so on. This might naively be interpreted as the idea that consciousness directly or indirectly alters the activity in the brain areas controlling speech, producing vibrations in the larynx that lead to sound vibrations in the air (see Figure 3).

The problem with this naive picture is that consciousness could only e-cause a chain of events leading to a verbal report if it could pass a physically conserved quantity, such as energy-momentum or charge, to neurons in the reporting chain—for example, if it could push them over their threshold and cause them to fire^18^. If the physical world is e-causally closed (A5), then a conserved quantity can only be passed from consciousness to an area of the brain if consciousness is a physical phenomena, i.e., if consciousness *is* the correlates of consciousness (C1 in Figure 3)^19^.

While it is possible that some version of identity theory or physicalism is correct, it would be controversial to base the scientific study of consciousness on this assumption, which would undermine our ability to gather data about the correlates of consciousness in a theory-neutral way. It would be much better if we could find a way of interpreting the measurement of consciousness that does not depend on the assumption that physicalism or functionalism are true.

In this paper it has been assumed that consciousness is functionally connected to the correlates of consciousness (L1), shown as C1 in Figure 3, and that c-reports contain information about all of the consciousness that is present. The only thing we need to fully account for the measurement of consciousness is a connection between C1 and the c-reports. This can be solved by introducing a further assumption that fits naturally within the current framework:

**A6**. The correlates of consciousness e-cause a platinum standard system's c-reports about consciousness.

This states that the correlates of consciousness are the first stage in a complex chain of e-causation that leads to c-reports about consciousness. Since it can be difficult to measure e-causation, in some circumstances A6 can be substituted for the weaker assumption:

**A6a**. The correlates of consciousness are effectively connected to a platinum standard system's c-reports about consciousness.

**Figure 4:**
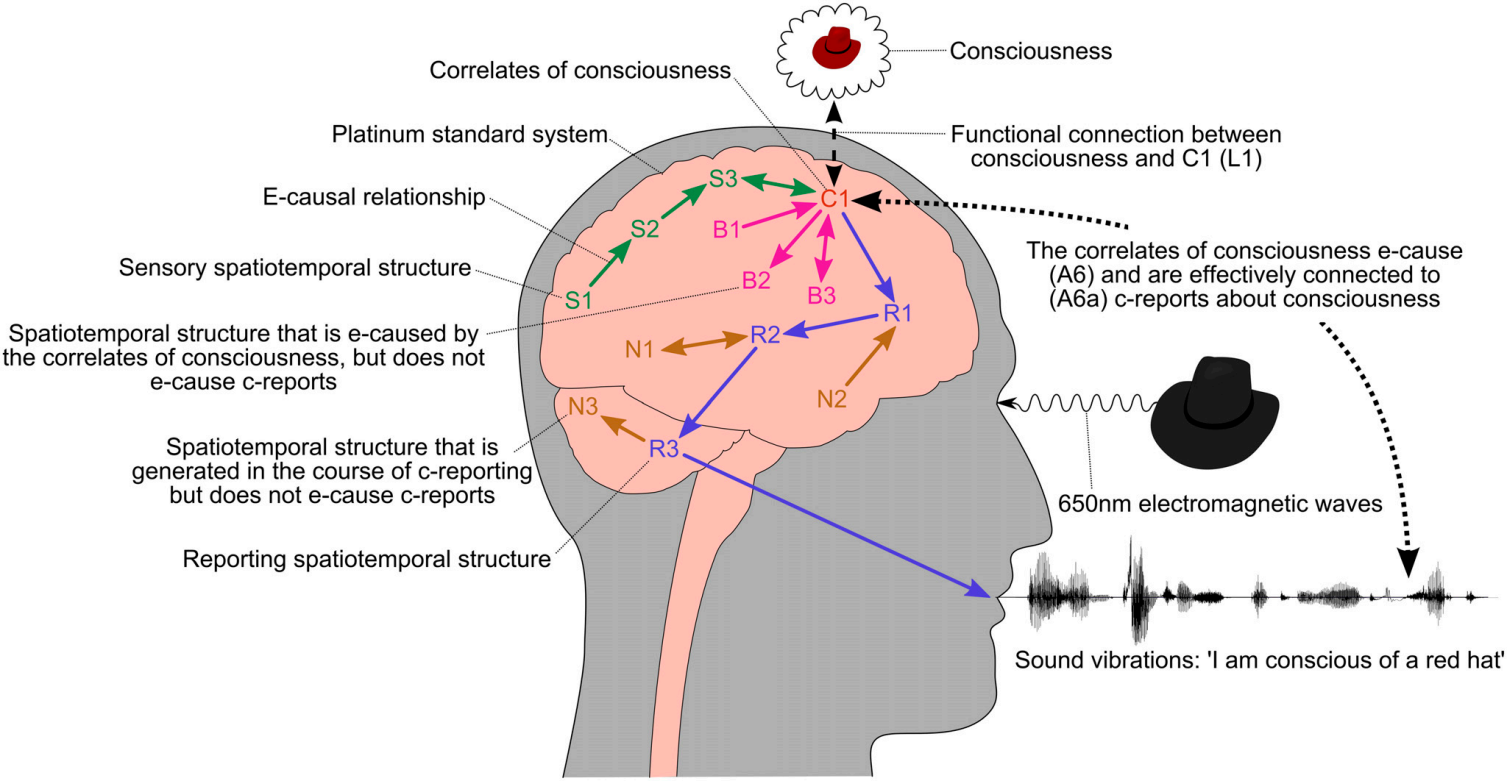
**The relationship between consciousness and c-reports about consciousness**. The labels S1, C1, R1 etc. refer to spatiotemporal structures in the brain, such as the activation of a brain area, neural synchronization, electromagnetic waves, quantum events, and so on. Their names and locations are only illustrative and not intended to correspond to particular anatomical paths or structures. S1–S3 are unconscious spatiotemporal structures that pass information to C1, such as the early visual stages leading up to and possibly including V1. The correlates of consciousness, C1, is a spatiotemporal structure in the brain that is assumed to be functionally connected to conscious states (L1). This could include sensory areas, such as MT/V5. C1 is assumed to be the first stage in an e-causal chain of spatiotemporal structures, R1–R3, that lead to a report about consciousness. B1–B3 and N1–N3 are spatiotemporal structures that are directly or indirectly connected to C1, but do not e-cause c-reports about consciousness.

Effective connectivity can be measured using algorithms, such as transfer entropy (Schreiber, 2000) or Granger causality (Granger, 1969)^20^, which work on the assumption that a cause precedes and increases the predictability of the effect. However, this does not always coincide with e-causation—for example when an unknown third source is connected to two areas with different delays. By themselves A6 and A6a do not say anything about the strength of the relationship between the correlates of consciousness and the c-reports about consciousness—for example, there could be a very weak e-causal chain leading from the correlates of consciousness to the c-reports, which could be primarily driven by unconscious brain areas. Assumptions A6 and A6a are illustrated in Figure 4.

We now have everything that we need for the measurement of consciousness during an experiment on the correlates of consciousness. During an experiment there is a functional connection between consciousness and C1. All of the consciousness is available for report (A4) and c-reporting does not break the causal closure of the physical world (A5) because the c-reports about consciousness are e-caused by C1 (A6).

### Two types of correlates of consciousness

It is hoped that experimental work will eventually identify a minimal set of spatiotemporal structures that are correlates of consciousness according to definition D1, because they are present when a particular conscious experience, *e*_1_, is present and absent as a collection when *e*_1_ is absent. According to assumption A6, these correlates should e-cause c-reports during experiments on the correlates of consciousness. However, it is possible that there are spatiotemporal structures in the brain that are correlates of consciousness according to D1, but cannot e-cause c-reports. This suggests that proposed correlates of consciousness can be divided into two types:

**Type A**. A spatiotemporal structure that matches definition D1 and can e-cause c-reports about consciousness. Type A correlates are plausible candidates for metaphysical theories of consciousness, such as physicalism, because the correlate actually e-causes the c-report.
**Type B**. A measurement of the physical system that is correlated with consciousness, but there is no plausible mechanism by which this correlate could e-cause c-reports about consciousness. This type of correlate might be an accurate predictor of consciousness, but consciousness cannot be claimed to be identical with this type of correlate because this would break the link between consciousness and c-reports. Type B correlates can be interpreted as indirect methods for identifying the presence of type A correlates.

Whether a correlate of consciousness is type A or B hinges on whether the correlate and its microphysical reduction [see section Empirical Causation (e-causation)] can be interpreted as e-causing c-reports about consciousness.

A clear example of a type A correlate is a functional correlate of consciousness, such as a global workspace, implemented in spiking neurons^21^. The macro-scale function can be reduced down to activity patterns in spiking neurons, which pass physically conserved quantities to other spiking neurons and can e-cause c-reports about consciousness. A clear example of a type B correlate would be a fMRI pattern that was correlated with consciousness. fMRI measures changes in blood flow in the brain, which can be used to infer the relative levels of neuron activity. While oxygen could be said to indirectly e-cause a neuron to fire, the fMRI measurement peaks several seconds after neurons have fired—indicating an influx of blood to replace oxygen depleted by recent activity. Since the fMRI signal can occur after a report about consciousness has been made, it cannot be an e-cause of c-reports.

More ambiguous examples of type B correlates are measures of causation, such as causal density (Seth et al., 2006) and liveliness (Gamez and Aleksander, 2011), which plausibly correspond to the rate of exchange of physically conserved quantities within a particular area. However, the amount of causal interaction within an area is dissociable from its causal interactions with other areas, which suggests that causal density and liveliness are not likely to be capable of e-causing c-reports. Similar issues apply to the information integration theory of consciousness (Tononi, 2008), since is not clear how a high level of information integration within a particular brain area could e-cause c-reports.

## Separating out the correlates of consciousness

This section briefly explores how the framework presented in this paper relates to experimental work on the neural correlates of consciousness. This has to distinguish the correlates of consciousness, from sensory and reporting structures that carry information to and from the correlates. The correlates also have to be separated from prerequisites and consequences that typically co-occur with consciousness. All of the labels for spatiotemporal structures in the brain (C1, S1–S3, R1–R3, etc.) are taken from Figure 4,^22^.

S1–S3 are unconscious sensory processing stages, such as activity in the visual system up to and possibly including V1. Later sensory processing stages, such as MT/V5, are likely to be included in the correlates of consciousness, C1. A common technique for separating S1–S3 from C1 is to present a constant stimulus to the subject that leads them to have alternating conscious experiences, which they report using a button press or similar behavior. The spatiotemporal structures in the brain that that covary with the reported consciousness are assumed to be part of C1, whereas spatiotemporal structures that remain tied to the sensory stimulus are assumed to be part of S1–S3 (de Graaf et al., 2012). Binocular rivalry experiments are typical examples of this type of work (Blake, 2001) and it can also be carried out using bistable images, such as a Necker cube. A second way of distinguishing S1–S3 from C1 is to measure the brain when the subject is perceiving an object and contrast this with the state of the brain when the subject is imagining or remembering the same object. The spatiotemporal structures that are common to the two situations are likely to be part of C1. A third approach is to use lesioning or TMS to selectively disable S1–S3 to establish whether they are correlated with conscious states. A fourth method is to present masked stimuli to the subject to identify the parts of the brain that process information unconsciously (Dehaene et al., 2001), and a fifth technique is to use the type of information that is processed in a particular brain area to determine whether it is part of C1. For example, Lamme (2010) suggests that the features of consciously perceived objects, such as color, shape, and motion, are bound together, whereas this type of binding is not present in the early visual system. The brain also contains a substantial amount of information that cannot be consciously accessed, such as the body-centric information used for motor control (Goodale and Milner, 1992). The spatiotemporal structures processing this type of information are unlikely to be part of C1.

Distinguishing C1 from R1–R3 is potentially problematic because information in C1 will appear in different forms along the e-causal chain from C1 to R3, and so measurements of any of these spatiotemporal structures could potentially be used to make predictions about consciousness. Another potential difficulty is that the communication mechanisms that facilitate c-reporting could be confused with the correlates of consciousness because they are present when consciousness is present and potentially absent when consciousness is absent. For example, neural synchronization might be an essential mechanism for any kind of reporting (communication through coherence) and have nothing to do with consciousness^23^. Some progress could be made by measuring the brain while the subject uses different methods to make the same c-report about consciousness. For example, they could verbally describe their consciousness, describe it using sign language, write down a description, describe it after short and long delays, and so on. Each of these c-reporting methods will involve different spatiotemporal structures in the brain, whereas the correlates of consciousness should be similar in each case. A second approach would be to accurately measure the timing of different events in the brain. It is expected that the correlates of consciousness should occur after the sensory chain S1–S3 and before R1–R3. A third method would be to use a backtracing procedure (Krichmar et al., 2005) that starts at the motor output stage and works back through the brain to locate the start of the reporting e-causal chain R1–R3. This should be halted just before it enters the unconscious sensory processing stages S1–S3.

B1–B3 roughly correspond to what de Graaf et al. (2012) and Aru et al. (2012) describe as the prerequisites and consequences of consciousness, although late stage sensory and early stage reporting could also be included in this category. B1 could be a mechanism that is necessary for the correlates of consciousness to occur, but is not actually correlated with consciousness. For example, a background level of activity, possibly provided by the reticular activating system, might be needed to bring the neurons in C1 closer to threshold so that the correlates of consciousness can take place, and it has been suggested that attention might be necessary for consciousness, but not directly correlated with it (de Graaf et al., 2012). In some cases B1 could be separated out by disabling it and C1 facilitated with a different method—for example, the reticular activating system could be disabled and a chemical added to C1 to bring the neurons closer to threshold. Some of the methods for dissociating S1–S3 from C1 could also be used to separate C1 from B1—for example, B1 might contain information that cannot be consciously accessed, which would suggest that it is not directly correlated with consciousness.

B2 is a spatiotemporal structure that is a consequence of C1, but which is not directly associated with consciousness and does not lead to a c-report. For example, a conscious image might activate unconscious representations or B2 could be an event related to memory consolidation (Aru et al., 2012). This type of spatiotemporal structure is relatively straightforward to distinguish from C1 because disabling it (lesion or TMS) should not affect consciousness or c-reports. The recurrent connection between C1 and B3 will make B3 difficult to separate out and it could easily be mis-identified as the source of the c-reports. If B3 is a prerequisite of C1, then a similar approach to B1 could be pursued, or B3 could be dissociated from C1 if it contained unconscious information. Other strategies for separating B1–B3 from C1 are discussed by de Graaf et al. (2012) and Aru et al. (2012).

N1–N3 are spatiotemporal structures that are generated in the course of c-reporting, but are not e-causes of the c-report. They need to be considered because backtracing methods could mistakenly identify N1 and N2 as e-causes of the c-report, and C1 might be effectively connected to N1 and N3. The use of different c-reporting mechanisms is likely to produce some progress with the dissociation of N1–N3 from C1, and many of the methods that have been suggested for S1–S3, R1–R3, and B1–B3 are applicable to N1–N3.

Depending on what C1 turns out to be there are likely to be multiple interpretations of what the actual correlates of consciousness are. For example, if C1 turned out to be a global workspace implemented in neurons synchronized at 40 Hz, then is the correlate some biological feature of the neurons, the function, the electromagnetic waves generated by the synchronization, or all of these together? A more detailed discussion of how different candidate correlates can and cannot be separated out is given by Gamez (2012).

In practice, the large amount of feedback between brain areas is likely to make the separation of the different spatiotemporal structures illustrated in Figure 4 extremely difficult. The different time scales on which different types of information are processed will complicate the picture, and the spatial and temporal resolution of our current measuring procedures are completely inadequate for the task. In the future optogenetic techniques might help to address some of these problems, and many of the difficulties can be understood by building models of proposed correlates of consciousness and examining how they can be distinguished from other spatiotemporal structures in the brain.

## Discussion and conclusions

This paper has put forward a set of assumptions that could account for our ability to measure consciousness through c-reports. This framework starts with the idea that c-reports about consciousness from platinum standard systems are functionally connected to the platinum standard systems' consciousness. This enables consciousness to be measured during experiments on the correlates of consciousness. Further assumptions were introduced to explain how consciousness could be connected to c-reports without breaking the causal closure of the physical world.

A person who accepts this framework can set aside philosophical debates about color inversion and zombies and focus on the empirical work of identifying correlations between consciousness and the physical world. Their measurement of consciousness will not be contingent on an acceptance of functionalism or physicalism, and it will not depend on an e-causal relationship between consciousness and the physical world. This framework prevents scientific results about consciousness from being undermined by philosophical problems: a scientist who rejects it will have to account for the measurement of consciousness in some other way.

While this framework has many benefits for scientific work on consciousness, it also imposes constraints. Results about the correlates of consciousness can only be considered to be true *given these assumptions*. Although this framework is compatible with most of the traditional metaphysical approaches to consciousness (for example, physicalism, dualism, and epiphenomenalism), it is not compatible with panpsychism and type B theories about the correlates of consciousness. Scientists who accept this framework will have to avoid panpsychist theories, and they should ensure that proposed correlates of consciousness are capable of e-causing c-reports during experiments on the correlates of consciousness. Information integration theories of consciousness are particularly problematic when considered in the light of these requirements since they propose that all information integration is associated with some level of consciousness, and it is not clear how information patterns could e-cause c-reports. The plausibility of other scientific theories of consciousness should be judged relative to these constraints.

This paper has assumed that consciousness is always potentially accessible during experiments on the correlates of consciousness (A4). Once the correlates of consciousness have been identified they could be used make predictions about inaccessible consciousness in non-experimental situations. An example of this type of reasoning can be found in Lamme (2006, 2010), who uses paradigmatic cases of reportable consciousness to establish the link between consciousness and recurrent processing, and then makes inferences about the presence of inaccessible phenomenal consciousness. Knowledge about the correlates of consciousness could also be used to make predictions about consciousness in systems that are not platinum standards, such as brain-damaged patients, infants, animals, and artificial systems.

Controversial experiments by Libet (1985) have indicated that our awareness of our decision to act comes after the motor preparations for the act (the readiness potential). This suggests that our conscious will might not be the cause of our actions, and Wegner (2002) has argued that we make inferences after the fact about whether we caused a particular action. These results could be interpreted to show that the correlates of consciousness do not e-cause c-reports about consciousness because motor preparations for verbal output (for example) would precede the events that are correlated with consciousness. This problem could be resolved by measuring the relative timing of a proposed correlate of consciousness (C1) and the sequence of events leading to the report about consciousness, including the readiness potential (R1–R3). If the framework presented in this paper is correct, then it should be possible to find correlates of consciousness that have the appropriate timing relationship; if no suitable correlates can be found, then the framework presented in this paper should be rejected as flawed^24^.

The basic illustration in Figure 4 shows incoming sensory data being transformed into a correlate of consciousness that e-causes c-reports. This would be questioned by people who see the brain as dynamically engaged with the world and are skeptical about internal representations—for example, O'Regan and Noë (2001) and Noë (2009). Sensorimotor theorists have also claimed that there is an identity between our sensorimotor engagement with the world and consciousness, which would make it necessary to include the body and environment in C1 and lead to a modification of assumption A1. Whatever the nature of C1 turns out to be, the e-causal relationship between C1 and R1–R3 has to be retained by any theory of consciousness that claims to explain how we can empirically study correlations between measurements of consciousness and the physical world.

It is reasonably easy to see how the contents of consciousness that are c-reported could be e-caused by physical events. For example, we can tell a simple story about how light of a particular frequency could lead to the activation of spatiotemporal structures in the brain, and how learning processes could associate these with sounds, such as “red” or “rojo.” This might eventually enable a trained brain to produce the sounds “I can see a red hat” or “I am aware of a red hat” when it is presented with a pattern of electromagnetic waves. Since consciousness does not appear to us as a particular thing or property in our environment and many languages do not contain the word “consciousness” (Wilkes, 1988), it is not necessary to identify sensory stimuli that the physical brain could learn to associate with the sound “consciousness.” The concept of consciousness can be more plausibly interpreted as an abstract concept that is acquired by subjects in different ways, and it is conceivable that the science of consciousness could be carried out without subjects ever using the word “consciousness” in their c-reports.

Like Chalmers' (1998) pre-experimental bridging principles, many of the assumptions set out in this paper cannot be experimentally tested because they are a condition of possibility of any kind of empirical work on consciousness. They can be seen as a preliminary attempt to shift the study of consciousness from a pre-paradigmatic state (Metzinger, 2003) to a paradigmatic science—an attempt to articulate the paradigm that will govern our normal scientific work on consciousness (Kuhn, 1970). Although many parts of this framework cannot be tested, its self-consistency can be improved as well as the way in which it relates to general principles in the philosophy of science and the study of consciousness. A science of consciousness based on it might also reach the point at which it no longer coherently hangs together, which might force us to abandon the scientific study of consciousness altogether or to formulate a completely new set of framing principles.

### Conflict of interest statement

The author declares that the research was conducted in the absence of any commercial or financial relationships that could be construed as a potential conflict of interest.

## Footnotes

^1^This paper will not consider whether the measurement of consciousness should involve the assignment of numbers to different aspects of it. The conception of measurement in this paper is more similar to the use of a level of abstraction to extract data sets that can have a wide range of types (Floridi, 2008), than to the use of numerical measurement scales, such as those discussed by Krantz et al. (2006).

^2^It is assumed that a single stream of consciousness is being reported. Many of the issues raised in this paper are also applicable to Dennett's (1991) multiple drafts model.

^3^See Sandberg et al. (2010) for a comparison of post-decision wagering, the perceptual awareness scale and confidence ratings.

^4^In the explication interview (EI) a trained person interviews a subject about a conscious experience to help them provide an accurate report (Petitmengin, 2006). In descriptive event sampling (DES) the subject carries a beeper, which goes off at random several times per day. When they hear the beep the subject makes notes about their consciousness just before the beep. This is followed by an interview that is designed to help the subject to provide faithful descriptions of the sampled experiences (Hurlburt and Akhter, 2006). Froese et al. (2011) discuss some of the first- and second-person methods for measuring consciousness.

^5^I selected the term “platinum standard system” as a reference to the platinum bar that was the first working definition of a meter. Other objects were directly or indirectly compared to this platinum bar to measure their length, but the length of the bar itself could not be checked because it was not meaningful to compare it with itself. In a similar way, the normally functioning adult human brain is a system that is our starting point for consciousness science. In this system consciousness is simply assumed to be present. Once we have established which spatiotemporal structures are correlated with consciousness in this “platinum standard” system, we can use our knowledge about these spatiotemporal structures to measure consciousness in other systems.

^6^A number of people have questioned the assumption that consciousness is correlated with brain states alone. For example, O'Regan and Noë claim: “There can therefore be no one-to-one correspondence between visual experience and neural activations. Seeing is not constituted by activation of neural representations. Exactly the same neural state can underlie different experiences, just as the same body position can be part of different dances” (O'Regan and Noë, 2001, p. 966). A less radical position can be found in Noë's later work: “A reasonable bet, at this point, is that some experience, or some features of some experiences, are, as it were, exclusively neural in their causal basis, but that full-blown, mature human experience is not” (Noë, 2004, p. 218). I have presented my own view on this issue elsewhere (Gamez, 2014). The implications that it might have for the scientific study of consciousness are discussed at the end of the paper.

^7^In previous work I have made the assumption that the *awake* normal adult human brain is a platinum standard system (Gamez, 2011, 2012). Assumptions A3 and A4 make the assumption that the brain is awake unnecessary—in the absence of c-reports, the brain is assumed to be unconscious.

^8^Functional connectivity (a deviation from statistical independence between A and B) is typically contrasted with structural connectivity (a physical link between A and B) and from effective connectivity (a causal link from A to B). A number of algorithms exist for measuring functional connectivity (for example, mutual information) and it can be measured with a delay. These algorithms cannot be used to measure the functional connectivity between consciousness and the c-reports because consciousness cannot be directly measured.

^9^This is expressed by Block (2007) as the idea of cognitive accessibility.

^10^Shanahan (2010) suggests how an omnipotent psychologist could extract data about consciousness in this way.

^11^I have argued elsewhere (Gamez, 2008) that Block's (1995) notion of “access consciousness” is better described as unconscious or non-conscious representational states, rather than as a form of consciousness.

^12^Chalmers (2000) distinguishes the total neural basis from the core neural basis: “A total NCC builds in everything and thus automatically suffices for the corresponding conscious states. A core NCC, on the other hand, contains only the ‘core’ processes that correlate with consciousness. The rest of the total NCC will be relegated to some sort of background conditions required for the correct functioning of the core” (Chalmers, 2000, p. 26). Block (2007) makes a similar distinction.

^13^If you think that “necessary and sufficient” doesn't imply causality, consider reversing the sentence “Neural synchronization is necessary and sufficient for consciousness.” Many people would consider “Consciousness is necessary and sufficient for neural synchronization” to be incoherent or wrong, which suggests that “necessary and sufficient” has causal overtones.

^14^These diagrams attempt to strike a balance between clarity and accuracy, which has led to a number of compromises. The most serious is that the brain is shown as pink, whereas in fact it is colorless (Metzinger, 2000). The depiction of a bubble of consciousness floating above a brain is purely metaphorical and I am not taking sides on the debate about the relationship between consciousness and representations.

^15^This is similar to Fell et al's. (2004) distinction between efficient and explanatory causation. Efficient causation is concerned with the physical relation of two events and the exchange of physically conserved quantities. Explanatory causation refers to the law like character of conjoined events.

^16^Kim (1998) gives a detailed discussion of the relationship between macro and micro physical laws.

^17^Kim makes a similar point about the causal closure of the physical world: “If you pick any physical event and trace out its causal ancestry or posterity, that will never take you outside the physical domain” (Kim, 1998; Kim, p. 40).

^18^Wilson (1999) discusses the minimum amount of physical effect that would be required for consciousness to influence the physical world. Burns (1999) gives a similar discussion in relation to the problem of free will.

^19^A related point is made by Fell et al. (2004), who argue that the neural correlates of consciousness cannot e-cause conscious states.

^20^Chicharro and Ledberg (2012) discuss the extent to which Pearl (2000) interventionist theory of causality could be used to measure causal relationships in the brain.

^21^See Gamez et al. (2013) and Zylberberg et al. (2010) for examples of such models.

^22^There is not space in this paper to provide a full summary of experiments on the neural correlates of consciousness. More detailed reviews are given by Rees et al. (2002); Tononi and Koch (2008), and Dehaene and Changeux (2011).

^23^Other issues related to the separation of consciousness from reporting mechanisms are discussed by Block (2007).

^24^It is worth noting that Libet's measurement of the timing of conscious events implicitly depends on a functional connection between consciousness and c-reporting behavior—the relative timing of consciousness and action can only be measured if consciousness is functionally connected to c-reports about consciousness.

## Appendix

### Definitions

**D1**. A platinum standard system is a physical system that is assumed to be associated with consciousness some or all of the time.
**D2**. A correlate of a conscious experience, *e*_1_, is a minimal set of one or more spatiotemporal structures in the physical world. This set is present when *e*_1_ is present and absent when *e*_1_ is absent.

### Assumptions

**A1**. The normally functioning adult human brain is a platinum standard system.
**A2**. The consciousness associated with a platinum standard system nomologically supervenes on the platinum standard system. In our current universe physically identical platinum standard systems are associated with identical consciousness.
**A2a**. The consciousness associated with a platinum standard system nomologically supervenes on the correlates of consciousness in the platinum standard system. In our current universe the spatiotemporal structures that correlate with conscious experience *e*_1_ will be associated with *e*_1_ wherever they are found.
**A3**. During an experiment on the correlates of consciousness, the consciousness associated with a platinum standard system is functionally connected to its c-reports about consciousness.
**A4**. During an experiment on the correlates of consciousness all conscious states associated with a platinum standard system are available for c-reports about consciousness.
**A5**. The physical world is e-causally closed.
**A6**. The correlates of consciousness e-cause a platinum standard system's c-reports about consciousness.
**A6a**. The correlates of consciousness are effectively connected to a platinum standard system's c-reports about consciousness.

### Lemmas

**L1**. There is a functional connection between consciousness and the correlates of consciousness.
